# Factors associated with congenital anomalies in Addis Ababa and the Amhara Region, Ethiopia: a case-control study

**DOI:** 10.1186/s12887-018-1096-9

**Published:** 2018-04-25

**Authors:** Molla Taye, Mekbeb Afework, Wondwossen Fantaye, Ermias Diro, Alemayehu Worku

**Affiliations:** 1 Department of Anatomy, School of Medicine, College of Medicine and Health Sciences, the University of Gondar, Gondar, Ethiopia; 2 Department of Anatomy, School of Medicine, College of Health Sciences, Addis Ababa University, Addis Ababa, Ethiopia; 3 School of Dentistry, College of Health Sciences, Addis Ababa University, Addis Ababa, Ethiopia; 4 School of Medicine, College of Medicine and Health Sciences, the University of Gondar, Gondar, Ethiopia; 5 School of Public Health, College of Health Sciences, Addis Ababa University, Addis Ababa, Ethiopia

**Keywords:** Congenital anomaly, Case-control, Teratogen, Risk/Associated factors

## Abstract

**Background:**

The early stage of embryo development is extremely vulnerable to various teratogenic factors, leading to congenital anomalies. In Ethiopia, a significant number of babies are born with congenital anomalies, but the risk factors for the anomalies have never been studied. Understanding the specific risk factors for congenital anomalies is very essential to provide health education that aims at creating awareness and establishing preventive strategic plan/s. The main objective of this study was to assess the risk factors associated with congenital anomalies in Addis Ababa and the Amhara Region, Ethiopia.

**Methods:**

A case-control study was conducted from January 1- June 30, 2015. The participants were recruited at the purposively selected hospitals in Addis Ababa and the Amhara Region. A total of 207 cases and 207 controls were included in the study. Cases were neonates, infants, and children 0-11 months of age with external and internal major congenital anomalies diagnosed by pediatricians. Controls were neonates, infants, and children 0-11 months of age without external and internal anomalies. Data on sociodemographic characteristics, exposure to risk factors, and reproductive history were collected by face to face interviews with children’s mothers/caregivers using a structured questionnaire. Binary logistic regression was employed to explore risk factors associated with the occurrence of the problems.

**Results:**

About 87.4% of the children were below 6 months, and 12.6% were between 6 and 11 months. The majority (59.9%) of the children were male, with the M: F sex ratio of 1.49. The mean age of the mothers was 26 years (16-45 years). Unidentified medication use during early pregnancy (AOR = 4.595; 95% CI: 1.868-11.301, *P*-value = 0.001), maternal alcohol drinking (AOR = 2.394; 95% CI: 1.212-4.726, *P*-value = 0.012), and exposure to chemicals (AOR = 9.964; 95% CI = 1.238-80.193, *P*-value = 0.031) were significantly associated with the occurrence of congenital anomalies. Iron folate use (AOR = 0.051; 95% CI: 0.010-0.260, *P*-value = < 0.001) before and during early pregnancy had a protective effect on congenital anomaly.

**Conclusion:**

Unidentified medication use, alcohol drinking during early pregnancy, and exposure to chemicals had a significant association with the occurrence of congenital anomalies, whereas iron folate use before and during early pregnancy had a protective effect from congenital anomalies.

## Background

The early stages of embryo development are a critical period which may mark vulnerability to disruption by teratogenic agents that lead to congenital anomaly [1–3]. Congenital anomaly (CA) is defined as a structural or functional defect that may be detected during pregnancy or be visible at birth or later in life. CAs are the major cause of new born/infant morbidity, mortality, and disability in addition to adding to the burden of the health care system [4]. It is estimated that about 20–30% of infant deaths occur due to CAs [5]; severe CAs occur in 3% of live [6, 7], and 20% of still births [8], and 7.9 million children are born with serious CAs, accounting for 6% of all births globally [9].

According to literature, genetic and environmental factors play a major role in causing CAs during the first trimester [10]. Approximately, 7.6 million children are born with CAs worldwide due to genetic factors [11]. Although there is a better understanding of the etiology of CA in the literature, the causes of most of the CAs are still unknown. However, they are believed to be caused by several etiologic factors [12, 13].

The occurrence of certain risk factors, such as maternal chronic diseases, like diabetes, family history, and viral infections during early pregnancy increase the risk of having a baby with CAs [14–17]. Furthermore, risk factors linked to the occurrence of CAs include unidentified medication/illicit/prescribed drug/s use, alcohol consumption, smoking cigarettes, exposure to radiation, chemical agents, parental age, parental occupation, nutritional deficiencies, and chromosomal mutations as well as socio-economic factors [7, 14, 15, 18–20]. Hence, exposure to all of these factors can influence the organogenesis processes of the embryo [7, 12].

Moreover, some studies showed that maternal use of folic acid and multivitamins containing folic acid around conception and the first 3 months of pregnancy have a protective effect against certain types of CAs [21, 22].

In general, the prevalence of CAs varies with race, ethnicity, and geographical regions across the world [7, 11, 16, 23, 24]. In Ethiopia, a significant number of babies are born with CAs, but the causes of these CAs have never been studied. In fact, whether the causes are environmental or genetic factors or interactions between environmental and genetic factors are not known. In addition, there are no preventive strategic plans to reduce or control the occurrence of these problems. It is obvious that children born with major CA are prone to die or even if they survive, they may confront a long-term morbidity and disability and may also undergo repeated surgical interventions. Evidence based information about specific risk factors for CAs are very essential to provide health education to communities, especially to females in the reproductive age. This could not only help to create awareness so as to reduce the occurrence of anomalies but also assists policy makers/responsible bodies to develop preventive strategic plans.

The main objective of this study was to assess the risk factors for CAs in Addis Ababa and the Amhara Region. The results are believed to provide information for public health actions and to create awareness about CAs and their etiologic factors. They may also be used as baseline for further investigations.

## Methods

### Study sites

The study was carried out in Addis Ababa and the Amhara region, Ethiopia.

Addis Ababa, the capital of Ethiopia, has 10 sub-cities with an estimated total population of 3,273,001(47.4% male, 52.6% female). The population of Addis is diverse by ethnicity. The majority of the people are Christians, followed by Muslims and some followers of other religions [25]. The city is subject to population increase through continuous migrations [25]. All of the hospitals in the city provide inpatient, outpatient, and delivery services, except Cure Ethiopia International Children’s Hospital, which does not provide delivery services.

The Amhara Region, the second largest northwest region of the country has a population of 20,399,004 (50.1% male, 49.9% female). The majority of the people live in rural areas and the minority in large and small towns. Most of the Amhara people are Christians, with a good number of Muslims and some followers of other faiths [25]. All of the hospitals in the regional state provide various inpatient and outpatient services including delivery.

### Selection of study hospitals

Five public hospitals in Addis Ababa and three in the Amhara Region were purposively selected for this study on the basis of case load. All of the hospitals are referral with high patient flow and various specialized departments or units, which provide various services to neonates, infants, children, and adults.

### Study design and sample size

A case-control design was used to investigate exposure status to risk factors for CAs in the case and control children.

A sample of 414 children (207 cases, 207 controls) was recruited for this study. The sample size was calculated by using a ratio of 1:1, power of 90%, a 5% significance level and 38.81% of likelihood of an event in controls (i.e. exposure in controls) [17], and assuming the Odds ratio to be 2. The study children were matched by age. Cases were neonates, infants, and children 0-11 months of age with external and internal major CAs. Major CAs are defined as anomalies that have partial or complete organ/organ system absence/defects, resulting in functional, health, and cosmetic effects, which require surgical repair and rehabilitations [4]. The cases were selected from live births at the study hospitals, neonate units and under five clinics. The type of CAs were classified as major by using WHO/CDC/ICBDSR manual 2014 [4]. Based on WHO/CDC/ICBDSR classification method, the cases with neural tube, orofacial region, musculoskeletal system and genitourinary system defects and less than 12 months of age were purposively selected and included in the study. Children aged 12 months and above and had major and minor CA were excluded. All cases had isolated CAs except one child who had multiple anomalies. The diagnoses of the cases were confirmed through physical examinations and echocardiography (i.e. for cases with heart defects) by pediatricians. The cases were selected purposively. The controls were neonates, infants, and children 0-11 months of age without external and internal CAs. The controls were randomly selected in the same hospitals where children with CAs were identified. All children aged 0-11 months constituted the source population.

### Data collection method

The data were collected by a face to face interview of the children’s mothers/caregivers by using a structured questionnaire and checklists. The questionnaire prepared in English and translated to Amharic was pretested at other hospitals and amended according to the results. Data were collected on residence, sociodemographic characteristics, reproductive/pregnancy history and adverse outcomes, family history of CAs, socio-economic status, parental ethnicity, parental occupation, maternal educational level, maternal nutritional status/history, parental exposures to risk factors and/or teratogenic agents, such as radiation, chemicals, pesticides, etc. for CAs, maternal illness/disease, medication/illicit drug use, substance abuse (alcohol consumption, smoking), blood relationships among parents, antenatal care visits, folic acid and multivitamin use, and birth order of children. The data were gathered at delivery rooms/wards, neonatal units, and under five clinics. Prior to data collection, data collectors (nurses, midwifes) were trained for 2 days. The data were collected from January 1- June 30, 2015.

### Data management and analysis

Data were checked on daily basis by collectors and further overseen by the principal investigator and supervisors for completeness and correctness during collection periods. It was also rechecked during data entry. The data were coded and entered into Epi-Info version 3.5.1 and then transferred to SPSS version 21 for analysis. Data cleaning, error checking, and analysis were conducted by using SPSS version 21. Data were calculated by using frequency, cross tabulation, and binary logistic regression. Significance level was considered at less than 0.05 *p*-value. Crude Odds ratio and confidence level were calculated. After the crude Odds ratios were identified, adjusted Odds ratios were computed to assess risk factors associated with the occurrence of CAs by using backward stepwise logistic regression. Exposure variables with *P*-value ≤0.2 in the crude Odds ratio were entered into the multivariable logistic regression model to observe their relationships and CAs (i.e. to observe associated variables with CAs in the adjusted Odds ratio). The characteristics of cases and controls were explained in percentages and numbers. The crude and adjusted Odds ratios, confidence intervals, *p*-values and the overall findings are presented in the result section in the form of texts and tables.

### Ethical clearance

Ethical clearance was obtained from Addis Ababa University, College of Health Sciences Institutional Review Board, the National Research Ethics Review Committee, Addis Ababa City Administration Health Bureau Ethical Clearance Committee, and the Amhara National Regional State Health Bureau Regional Health Research Laboratory Center. Supportive letters were written to all zonal health departments and study hospitals by the health bureaus. Ethical and supportive letters were submitted to all hospital administrators. The purpose of the study was explained to the participant children’s mothers/caregivers. Data collection began after permissions were obtained from hospital managers/medical directors and after a written consent was obtained from children’s mothers/caretakers. Data collected from the participants were kept in secured and locked cabinets in order to maintain confidentiality.

## Results

### Socio-demographic characteristics of the study participants

The sample consisted of 414 (207 cases, 207 controls) children. The age of children ranged from 1 day to 11 months. About 87.4% of the children were below 6 months and 12.6% between 6 and 11 months. The majority (59.9%) of the children were male and 40.1% female with a sex ratio of 1.49, respectively. The mean age of the children’s mothers was 26 years (range 16-45). Among the participants, 63.3% were urban and 36.7% rural dwellers. In terms of income, 47.8% had middle and 37.2% low economic status as stated by the mothers/respondents. The majority (93.5%) of the children’s mothers were married; about 37.4% had no formal education; 31.9% had primary education, and 30.7% secondary and higher level of education. By religious affiliation, 72.2, 27.3, and 0.5% of the parents were Christian, Muslim, and others, respectively. Among the mothers, 53% were housewives, 17.9% government, private, or self-employed, 14.3% farmers, and 14% jobless. Similarly, 49.3, 38.9, and 11.8% of the children’s fathers were employees, farmers, and jobless, respectively. The major (62.1%) ethnic group in this study were Amhara, 23.2% Oromo, 5.6% Guragie, 3.6% Tigre, and 3.6% Seltie. The other socio-demographic characteristics of the participants are shown in Table 1.

**Table 1:** The socio-demographic characteristics of the study participants, Addis Ababa and the Amhara Region, Ethiopia, 2016

Characteristics		Cases (*n* = 207)		Controls (*n* = 207)		Total (*n* = 414)	
		Frequency	%	Frequency	%	Frequency	%
Study Site	Addis Ababa	106	51.2	107	51.7	213	51.4
	Amhara Region	101	48.8	100	48.3	201	48.6
Infant/Child Age	Below 6 months	181	87.4	181	87.4	362	87.4
	6-11 months	26	12.6	26	12.6	52	12.6
Infant/Child Sex	Male	123	59.4	125	60.4	248	59.9
	Female	84	40.6	82	39.6	166	40.1
Child Mother Age	< 20 years	11	5.3	11	5.3	22	5.3
	20-34 years	167	80.7	169	81.6	336	81.2
	> 34 years	29	14.0	27	13.0	56	13.5
Child Mother Marital status	Married	190	91.8	197	95.2	387	93.5
	Never married	17	8.2	10	4.8	27	6.5
Religion	Christian	139	67.1	160	77.3	299	72.2
	Muslim	66	31.9	47	22.7	113	27.3
	Other	2	1.0	0	0	2	0.5
Child Mother Education	No education	101	48.8	54	26.1	155	37.4
	Primary	59	28.5	73	35.3	132	31.9
	Secondary and higher	47	22.7	80	38.6	127	30.7
Monthly income	Regular	84	40.6	74	35.7	158	38.2
	Irregular	123	59.4	133	64.3	256	61.8
Child Mother Ethnicity	Oromo	63	30.4	33	15.9	96	23.2
	Amhara	122	58.9	135	65.2	257	62.1
	Guragie	7	3.4	17	8.2	24	5.8
	Others^a^	15	7.3	22	10.6	37	8.7
Child Mother Occupation	House wife	122	58.9	101	48.8	223	53.9
	Employed	28	13.5	46	22.2	74	17.9
	Farmer/Agriculture	32	15.5	27	13.0	59	14.3
	No job/unemployed	25	12.1	33	15.9	58	14.0
Residence	Urban	97	46.9	165	79.7	262	63.3
	Rural	110	53.1	42	20.3	152	36.7
Economic Status	Low	89	43.0	65	31.4	154	37.2
	Middle	100	48.3	98	47.3	198	47.8
	higher	18	8.7	44	21.3	62	15.0
Child Father Ethnicity	Oromo	63	30.4	33	15.9	96	23.2
	Amhara	116	56	133	64.3	249	60.1
	Guragie	8	3.9	15	7.2	23	5.6
	Others^a^	20	9.6	26	12.6	46	10.9
Child Father Occupation	Employed	72	34.8	132	63.8	204	49.3
	Farmer/Agriculture	107	51.7	54	26.1	161	38.9
	No job/unemployed	28	13.5	21	10.1	49	11.8
Region originated	Amhara	106	51.2	101	48.8	207	50.0
	Oromia	45	21.7	18	8.7	63	15.2
	Addis Ababa	35	16.9	80	38.6	115	27.8
	Other regions ^b^	21	10.3	8	3.9	29	7.0

^a^Others = Tigre, Somali, Afar, Seltie, Benishangul, Agew, Gamo, Hadya, Argob

^b^Other regions = Southern Nations Nationalities and People, Tigray, Afar, Benishangul-Gumuz, Harari, Dire Dawa

About 12 (5.8%) and 13 (6.3%) of mothers whose children had and had no CAs, respectively, were in the 15-19 years of age group. Besides, 49 (23%) and 68 (32.9%) of mothers whose children were exposed and not exposed to CAs, respectively, were 20-24 years of age. Similarly, 69 (33.3%) and 68 (32.9%) of the mothers who had children with and without CAs, respectively, were in the 25–29 years of age group, whereas 49 (23.7%) and 31 (15%) mothers who had and free children, respectively, were in the 30–34 years of age group. Likewise, 28 (13.5%) and 27 (13%) of the mothers who had affected and non-affected children, respectively, were 35–39 years of age.

About 99.5% of the CAs identified were isolated/single, while 1 (0.5%) was multiple. The frequency of CAs by organ/organ system is shown in Table 2.

**Table 2:** Frequency of congenital anomalies by organ/organ system among the study subjects, Addis Ababa and the Amhara Region, Ethiopia, 2016

Type of Congenital Anomalies	frequency	%
Neural Tube Defects (Spina bifida with and without hydrocephaly, clubfoot; encephalocele (occipital, frontal, and nasal))	124	30
Orofacial Clefts (cleft lip, cleft lip and palate, and cleft palate)	54	13
Masculoskeletal Defects (reduction defects of tibia, femur, ulna, and radial bones, talipes equinovarus, tracheoesophageal fistula, gastroschisis, and amelia of upper limb)	20	4.8
Heart Defects (ventricular septal defects)	5	1.2
Genitourinary Anomalies (hypospadias with sub-coronal and penile)	3	0.7
Bladder extrusion with cleft lip and palate	1	0.2
No Congenital Anomalies/Controls	207	50
Total	414	100

### Reproductive, obstetric, and offspring background/history

As far as the birth order of children was concerned, 32.9, 19.8, and 47.3% were first, second, third and above (3+) children to their families, respectively. Among the controls, about 48.3, 22.7, and 29% were first, second, third and above (3+) children, respectively, to their parents. About 16.4% of mothers whose children had CAs and 9.2% of the mothers who had children without CAs, had no antenatal care (ANC) visits. The total number of pregnancy history, preterm, miscarriage, stillbirth, gravidity, gestational age at first ANC, number of live births, and neonate/infant/child deaths are presented in Table 3.

**Table 3:** Reproductive history of the study participants/children’s mothers, Addis Ababa and the Amhara Region, Ethiopia, 2016

Characteristics		Cases		Controls		Total	
		(*n* = 207)	%	(*n* = 207)	%	(*n* = 414)	%
Birth order	First	68	32.9	100	48.3	168	40.6
	Second	41	19.8	47	22.7	88	21.3
	Third and above	98	47.3	60	29.0	158	38.2
Total number of pregnancy	1	67	32.4	100	48.3	167	40.3
	2-3	69	33.3	70	33.8	139	33.6
	4 and above	71	34.3	37	17.9	108	26.1
Full and post term pregnancy	Post term	12	5.8	5	2.4	17	4.1
	1-2	124	59.9	165	79.7	289	69.8
	3 and above	71	34.3	37	17.9	108	26.1
Preterm	None	158	76.3	178	86.0	336	81.2
	1	47	22.7	26	12.6	73	17.6
	2 and above	2	1.0	3	1.4	5	1.2
Elective termination	None	184	88.9	199	96.1	383	92.5
	1-2	23	11.1	8	3.9	31	7.5
Miscarriage	None	131	63.3	174	84.1	305	73.7
	1-2	76	36.7	31	15.0	107	25.8
	3 and above	0	0	2	1.0	2	0.5
Still birth	None	164	79.23	190	91.8	354	85.5
	1-2	42	20.29	17	8.2	59	14.3
	3 and above	1	0.5	0	0	1	0.2
Neonate/Infant/child death	None	178	86.0	189	91.3	367	88.9
	1-2	29	14.0	17	8.2	46	11.1
	3 and above	0	0	1	0.5	1	0.2
Living children	None	7	3.4	0	0	7	1.7
	1-2	129	62.3	169	81.6	298	72.0
	3 and above	71	34.3	38	18.4	109	26.3
Gravidity	Primigravida	70	33.8	100	48.3	170	41.1
	Multigravida	137	66.2	107	51.7	244	58.9
Gestational age at 1st ANC (antenatal care) visit	No ANC visit	34	16.4	19	9.2	53	12.8
	1-3 months	27	13.0	29	14.0	56	13.5
	4 months and above	146	70.5	159	76.8	305	73.7
Institution for ANC attended	Public hospital	27	13.0	23	11.1	50	12.1
	Public health center	130	62.8	147	71.0	277	66.9
	Private hospital	6	2.9	4	1.9	10	2.4
	Private clinic	7	3.4	13	6.3	20	4.8
	Health post	3	1.4	1	0.5	4	1.0
	No ANC visit	34	16.4	19	9.2	53	12.8

### Risk factors associated with congenital anomalies

There were maternal/medical illnesses (i.e. infections or chronic diseases such as diseases with fever, chronic disorders) in 24 (11.6%) of the mothers who had children with CAs and 13 (6.3%) of mothers whose children were not affected with CAs. Passive smokers were observed in 37 (17.9%) and 28 (13.5%) of mothers who had children with and without CAs, respectively. Among the participants, unidentified medication use in early pregnancy by mothers whose children had and had no CAs were 16.9 and 3.9%, respectively. Contraceptive (both pill and injection) use around conception was 11.6% in the mothers who had children with CAs and 2.4% in the mothers who had no children with CAs. In addition, anemia was observed in 55 (26.6%) of the affected children’s mothers and 37 (17.9%) of mothers whose children had no CAs (Table 4).

**Table 4:** Participant’s response on risk factors for congenital anomaly: Results from multivariable analysis-adjusted for substance abuse/behavioral, parental, and environmental factors, maternal illness, iron folate/folic acid, multivitamin, and vegetable use variables, Addis Ababa and the Amhara Region, Ethiopia, 2016

Characteristics		Cases		Controls		COR	AOR	95% CI		*P*-value
								lower	upper	
		Number	%	Number	%					
Iron folate/folic acid use	Yes	2	1	22	10.6	0.082	0.051	0.010	0.260	< 0.001
	No	205	99	185	89.4	1Ref				
Unidentified medication use	Yes	35	16.9	8	3.9	5.062	4.595	1.868	11.301	0.001
	No	172	83.1	199	96.1	1				
Drank alcohol (daily, occasionally and 1-3times in a week)	Yes	49	23.7	18	8.7	3.256	2.394	1.212	4.726	0.012
	No	158	76.3	189	91.3	1				
Exp. to chemical (mother)	Yes	13	6.3	1	0.5	13.804	9.964	1.238	80.193	0.031
	No	194	93.7	206	99.5	1				
Contraceptive use	Yes	24	11.6	5	2.4	5.298	4.840	1.647	14.224	0.004
	No	183	88.4	202	97.6	1				
Blood relationship between child parents	Yes	10	4.8	2	1	5.203	6.234	0.959	40.521	0.055
	No	197	95.2	205	99	1				
Family history	Yes	9	4.3	3	1.4	3.091	3.397	0.862	13.393	0.081
	No	198	95	204	98.6	1				
Vegetable Serving	Yes	139	67.1	164	79.2	0.536	0.644	0.392	1.059	0.083
	No	68	32.9	43	20.8	1				
Maternal illness	Yes	24	11.6	13	6.3	1.957	2.025	0.903	4.54	0.087
	No	183	88.4	194	93.7	1				
Exp. to radiation before conception (child father)	Yes	7	3.4	2	1	3.587	6.838	0.741	63.109	0.090
	No	200	96.6	205	99	1				

*Exp* exposure, *COR* crude odds ratio, *AOR* adjusted odds ratio, *CI* confidence interval, *Ref* reference

Blood relationships among parents were reported in 4.8% of children’s the exposed mothers and 1.0% of not exposed children’s mothers. Family history of CA as seen in the children was also reported in 4.3% of the affected children’s parents and 1.4% of the parents whose children had no CAs. Exposure to chemicals among mothers who had children with and without CAs was 6.3 and 0.5%, respectively. On the other hand, two (0.97%) of mothers whose children had CAs used fertility enhancing drugs. Again, one (0.5%) of the affected child mother had diabetes during pregnancy. On the contrary, mothers who had children without CAs reported no use of fertility enhancing drugs around conception and had no diabetes during and before pregnancy. In addition, none of the affected children’s mothers took multivitamins before they got pregnant, while one (0.5%) of the mother who had child without CA used multivitamin before pregnancy. Moreover, about 25 (12.1%) of mothers whose children had CAs and 17 (8.2%) of the mothers who had children without CAs, had no food shortage before and during pregnancy (Table 4). However, the above-mentioned factors were not statistically significant in the multivariable analysis, the adjusted Odds Ratio. In addition, maternal age, parental ethnicity, mothers educational level, parental occupation (Table 1) and parity, birth order, preterm, still birth, and miscarriage (Table 3) were not statistically significant in the crude and adjusted Odds Ratio analysis.

Thirteen variables were entered into the COR analysis. Of these, unidentified medication used during the first 3 months of gestation (COR = 5.062; 95% CI: 2.286-11.206, *P*-value = < 0.001), alcohol consumption during early/throughout pregnancy (COR = 3.256; 95% CI: 1.823-5.816, *P*-value = < 0.001), anemia during early pregnancy (COR = 1.663; 95% CI: 1.038-2.662, *p*-value = 0.034), contraceptive use around conception (COR = 5.298; 95% CI: 1.980- 14.175, *P*-value = 0.001), and chemical exposure during early pregnancy (COR = 13.804; 95% CI; 1.789-106.522, *P*-value = 0.012), and blood relation among parents (COR = 5.203; 95% CI:1.126-24.046, *P*-value = 0.035) were associated with CAs in the crude Odds ratio analysis. On the other hand, folic acid/iron folate (COR = 0.082; 95% CI: 0.019-.354, *P*-value = 0.001) and vegetable servings/use during early pregnancy (COR = 0.536; 95% CI: 0.344-0.835, *P*-value = 0.006) was protective against the occurrence of CAs (Table 4). However, vegetable serving was not associated with the occurrence of CAs in the adjusted Odds ratio analysis.

Twelve variables (alcohol drinking, anemia, iron folate/folic acid, unidentified medication, contraceptive, exposure to chemicals, vegetable serving, blood relationship between child parents, exposure to radiation, maternal illness, family history, and food shortage during early pregnancy) whose value ended up with 0.2 and below in the crude Odds ratio were entered into the multivariable logistic regression model to observe exposure variables associated with CAs. As indicated in Table 4, unidentified medication use (AOR = 4.595; 95% CI: 1868-11.301, *P*-value = 0.001), maternal alcohol drinking in early pregnancy (AOR = 2.394; 95% CI: 1.212-4.726, *P*-value = 0.012), and exposure to chemicals during early pregnancy (AOR = 9.964; 95% CI: 1.238-80.193, *P*-value = 0.031) were significantly associated with the occurrence of CAs. On the contrary, iron folate/folic acid supplementation (use) before and during early pregnancy (AOR = 0.051; 95% CI: 0.010-0.260, *P*-value = < 0.001) had a protective effect against CA.

## Discussion

According to our findings, unidentified medication use, alcohol consumption during early pregnancy or throughout pregnancy, and exposure to chemicals during early pregnancy had significant associations with the occurrence of CAs in this study, whereas iron folate supplementation before and during early pregnancy showed a protective effect against CA.

Like our study, scientific literature reports that drug usage during pregnancy is associated with CAs [26–31]. In Ethiopia, drugs are being sold by private drug venders, stores, clinics, and some shops with and without prescriptions. This indicates that mothers are likely to use medications/over-the counter drugs during their early pregnancy. Therefore, responsible bodies should control over-the-counter.

The association of alcohol drinking during and before early pregnancy and CA was also reported by Grewal et al., [32], Zhang et al., [33], and O’Leary et al., [34, 35]. In the liver, alcohol is metabolized into acetaldehyde, a toxic chemical/substance. To convert this toxic substance into a non-toxic and usable chemical, enzyme acetaldehyde dehydrogenases that secreted converts the acetaldehyde into acetic acid, which is useable by the body. However, when the unconverted acetaldehyde accumulates in the body, it causes irritation of the cells and tissues and then damages the cells and tissues, leading to metabolism and cellular growth abnormalities [36]. Moreover, alcohol is capable of crossing the placenta membrane and being carried to all developing cells and tissues of the embryo and fetus, and as a result causes damages to the developing tissues and cells and subsequently leads to a structural abnormality. Furthermore, alcohol drinking during pregnancy results in a serious consequences which manifest with recognized characteristics of physical and mental abnormal features after a baby is born [36–44].

In Ethiopia, alcohol is consumed by the community [45, 46], particularly locally made alcoholic beverages known as “areki” and “tella” in Amharic are consumed by both men and women in rural communities, especially among Christians, more specifically Orthodox Christians, across the country, whereas some urban dwellers, including women drink beer and other alcoholic drinks. In this study, women who consumed alcohol daily and women who took alcohol to an increasing degree had children with CAs. Therefore, health education is essential for the community, particularly women in the 15-49 years of age group.

In the present study, exposure to chemicals had a significant association with CAs. Likewise, exposure to chemicals has been reported as having a significant association with the occurrence of CAs [47–51].

In this study, mothers of cases who reported maternal/medical illnesses during early pregnancy had higher odds of babies with CAs compared to mothers of controls. Similarly, maternal diseases or medical illnesses, such as syphilis, Rubella, measles, other viral infections, [52–56] and chronic diseases were reported by several researchers as known causes of CAs [14, 18, 52–59].

Many studies reported that iron folate/folic acid supplementation in the early pregnancy and before protected or reduced the occurrence of certain CAs. For example, neural defects can be reduced if a pregnant woman appropriately takes the recommended dose of iron folate/folic acid. Women who take iron folate/folic acid are less likely to have babies with CA compared to those who do not take folic acid during and before early pregnancy [14, 60–68]. Folic acid together with vitamin B12 plays a vital role in the synthesis of nucleic acid, lipids, and proteins, required for cell division. Iron folate/folic acid also has a role in amino acids metabolisms that are needed for DNA and RNA synthesis and plays an important role as an antioxidant agent. Therefore, it is essential to make it available to all pregnant women.

Various studies suggested that multivitamin or folic acid containing multivitamin use during/ before pregnancy reduces/protects against some CAs [60, 65–68]. Multivitamins are essential organic substances, which are required by the body for normal body metabolic functions, body building, and controlling chemical reactions, such as converting food substances into energy and living tissues, as well as for survival or living healthy. Some components of multivitamins act as antioxidant substances (i.e. protect cells from damage by free radicals), for example, vitamin “E” and “C”. Moreover, some components of multivitamins are also important for body defense mechanisms, for instance, zinc, vitamin “C”, and vitamin “A” protect the body from diseases.

As observed in this study, in Ethiopia, health care providers might not have been providing folic acid or multivitamin containing folic acid supplementation to pregnant women until recent times. However, recently the Federal Ministry of Health recommended iron folate supplementation to all pregnant women although most such women have not been provided still. Moreover, research on folic acid supplementation and knowledge of women about folic acid use during pregnancy is limited in Ethiopia.

In this study, maternal age, ethnicity, sex of child, maternal education, and parity were not significantly associated with the occurrence of CAs. This finding is in agreement with that of a study conducted in Saudi Arabia by Salih et al., [18]. However, previous studies have shown a linkage to maternal age, (greater than 35) [14, 69], maternal education, and parity with the occurrence of CA [69].

In the present study, cigarette smoking and passive smoking were not associated with CAs although several researchers reported that smoking/passive smoking was associated with a specific types of CAs [32, 70–73]. In some societies, women in the reproductive age group smoke cigarettes. This may be due to cultural and personal habits. In addition, exposure to radiation had no statistically significant link in this study. Moreover, blood relationship, family history, and the Odds of having children with CA had no statistically significant associations in this study. To the contrary, other studies reported that a significant association occurred between blood relationship/family history and CA [18, 73–75]. This variation might be due to differences in the cultures of consanguinity marriage and genetic makeup. In addition, there were no associations between birth order, preterm, miscarriage, and CAs in the present study.

In general, the present study indicates that the characteristics of cases and controls are closer, except for a few variations, like maternal alcohol drinking and maternal illness. In addition, our findings are in line with some previous reports and at variance with others. The reason they are different from the reports of some studies may be differences in socio-demographic, geographic, study setting, methodological, and environmental characteristics.

## Conclusion

The findings of this study showed that alcohol drinking, unidentified medication use, and exposure to chemicals had significant associations with the occurrence of CAs. Iron folate use before and during pregnancy indicated a protective effect on CAs. However, current iron folate and multivitamin supplementation to pregnant women has been inadequate and infrequent because only very few women are observed using them during pregnancy, while the majority of women in the reproductive age groups are not getting prior to conception and during early pregnancy. Therefore, we emphasize that the supplementation is required to reproductive age groups, particularly around conception and during early pregnancy.

Although the causes of CAs are unknown and not studied in Ethiopia, screening nutritional deficiencies, chronic diseases, such as anemia, diabetics, infections/diseases (i.e. viral infections and sexually transmitted diseases) and establishing preventive measures, for example, health education to the public, specifically to reproductive age women are very important. So, to understand and identify the risk factors for CAs, it is necessary to conduct further population based investigations in different parts of the country to further ascertain the presently observed risk factors in association with CAs, as well as fully explore factors, which are not associated with CAs.

## Abbreviations

ANC: Antenatal care
CA: Congenital anomaly
CAs: Congenital anomalies
WHO/CDC/ICBDSR: World Health Organization/Centers for Disease Control and Prevention/International clearinghouse for Birth Defects Surveillance and Research
